# The Manual Habituation and Discrimination of Shapes in Preterm Human Infants from 33 to 34+6 Post-Conceptional Age

**DOI:** 10.1371/journal.pone.0009108

**Published:** 2010-02-09

**Authors:** Fleur Lejeune, Frédérique Audeoud, Leïla Marcus, Arlette Streri, Thierry Debillon, Edouard Gentaz

**Affiliations:** 1 Laboratoire Psychologie et NeuroCognition (UMR CNRS), CNRS and Université Pierre Mendès France, Grenoble, France; 2 Service de Néonatologie, CHU Grenoble, Grenoble, France; 3 Laboratoire de Psychologie de la Perception (UMR CNRS), CNRS and Université Paris Descartes, Paris, France; University of Rennes 1, France

## Abstract

**Background:**

Grasping at birth is well-known as a reflex in response to a stimulation of the palm of the hand. Recent studies revealed that this grasping was not only a pure reflex because human newborns are able to detect and to remember differences in shape features. The manual perception of shapes has not been investigated in preterm human infants. The aim of the present study was to investigate manual perception by preterm infants.

**Methodology/Principal Findings:**

We used a habituation/reaction to novelty procedure in twenty-four human preterm infants from 33 to 34+6 post-conceptional age. After habituation to an object (prism or cylinder) in one hand (left or right) in a habituation phase, babies were given either the same object or the other (novel) object in the same hand in a test phase. We observed that after successive presentations of the same object, a decrease of the holding time is observed for each preterm infant. Moreover, a significant increase of the holding time is obtained with the presentation of the novel object. Finally, the comparison between the current performance of preterm infants and those of full-term newborns showed that preterm babies only had a faster tactile habituation to a shape.

**Conclusion/Significance:**

For the first time, the results reveal that preterm infants from 33 to 34+6 GW can detect the specific features that differentiate prism and cylinder shapes by touch, and remember them. The results suggest that there is no qualitative, but only quantitative, difference between the perceptual abilities of preterm babies and those of full-term babies in perceiving shape manually.

## Introduction

This research addresses the question of the ability of preterm babies to perceive various shapes manually. It is well-known that human beings possess tactile sensitivity from the first weeks of fetal life. Using a fine-haired esthesiometer to stroke the skin, Hooker (1938) and Humphrey (1964, 1970) were able to trigger fetal reactions and describe precisely the tactile sensitivity of the fetus's body [1], [2], [3]. The parts of the body which react to tactile stimulation are the area around the mouth (8.5 weeks), a reaction corresponding to an opening of the mouth and swallowing, the genital area (10.5 weeks), the palms of the hands (between 10.5 and 11 weeks), and the soles of the feet (12 weeks). Regarding manual skills, the observation of babies' motor behavior has contributed considerably to fixing developmental stages [4], [5]. These studies were not concerned with the fact that, contrary to those above, they disregard the newborn's potential, however weak, to gather information about objects and thus to perceive. Already present *in utero* [6], the grasping reflex in response to a stimulation of the palm of the hand was regarded as the newborn's and the infant's dominant behavior that favored a form of interaction with its environment. Pressure exerted on the palm of the hand by an object or even the observer's finger triggers the closing of the fingers around the stimulus. The avoiding reflex is added to the neonatal grasping reflex. This consists, on the contrary, in the opening wide of the fingers and the rejection of all stimulation [7], [8]. But, the avoiding reflex becomes dominant at 5 months of age. The presence of both reflexes at birth is regarded as the prelude to more voluntary actions, such as taking and releasing objects.

However, for a long time researchers were unaware of a third action taking place between the other two, namely the holding of objects. During holding, manipulation of the objects would allow the baby to gather information about them. In a similar way to the grasping reflex of the palm, oral pressure exerted on a bottle teat or a nipple was rightly regarded as the outward sign of the sucking reflex, whose main function was to allow the baby to feed itself. Extended to non-nutritive sucking situations, the unique function of this reflex was to calm the baby down and stop it from crying. The study of the active touch via hand, considered in isolation, allows us to answer questions about the development of manual shape perception by babies. Recent studies revealed that the grasping at birth is not only a pure reflex, because full-term newborns are able to discriminate different properties of objects with their hands, like weights [9], textures [10], [11], and substances [12]. Using a classic habituation/reaction to novelty procedure (without visual control), Streri, Lhote and Dutilleul (2000) showed that newborns are able to memorise tactile information about specific shape features (prism or cylinder) and detect differences between these two shapes with either the right or left hand [13].

Essentially, studies about preterm babies and touch concern pain and developmental cares [14].They revealed that neonates' pain responses are influenced by the number of painful procedures previously experienced by the infant [15]. Bartocci, Bergqvist, Lagercrantz and Anand (2006) showed that tactile and painful stimuli specifically activated somatosensory cortical areas [16]. This result indicates that there is a central integration of tactile information in preterm newborns at 28–36 weeks of gestation. The link between hand movements and somatosensory cortical activation has also been shown in preterm newborns at 29–31 weeks of gestation [17]. In addition, Kostovic and Jovanov-Milosevic (2006) showed that the organization of cerebral connections in the preterm infant is substantially different from that in newborns, giving evidence of the immaturity of the preterms' brain [18]. As a result, developmental cares have been elaborated. It is a method that provides a preventive and integrative approach for minimizing neonatal discomfort by promoting the infant's own regulatory capacities to cope with stress (for example the Newborn Individualized Developmental Care and Assessment program (NIDCAP) [19]). Early intervention could influence tactual abilities in preterm newborns. In fact, other studies examined passive touch or tactile sensitivity in preterm babies in comparison with full-term babies [20], [21]. These studies showed that full-term and preterm babies differ in behavior and cardiac responses to tactile stimulation when tested at comparable post-conceptional age. Furthermore, intervention (multimodal stimulations) seemed to reduce this difference showing that sensory experiences benefits preterm babies' development.

Nonetheless, little is known about preterm neonates and their active touch. The role of active touch is crucial to gathering information about objects and obtaining a better perception of objects' properties like information about shape. Taken together, behavioral and neurological data suggest that all preterm babies could have a relatively mature sense of touch at birth and thus the ability to perceive various shapes with each hand.

The main purpose of this study was to investigate the ability of preterm babies to perceive with one hand the difference between two shapes. We performed a classic habituation/reaction to novelty procedure without visual control (see *Procedure*). If, after successive presentations of the same object, preterm babies take in information with their hands, a decrease of the holding time should be observed, indicating a habituation process. Moreover, if they are able to discriminate between various shapes (prism vs. cylinder), then we expect a significant increase of the holding time for the presentation of the novel object during the test phase. Finally, data from Streri *et al*'s study (2000) [13] were compared with the present data in order to investigate whether these perceptual manual abilities were qualitatively and/or quantitatively different between preterm and full-term newborns.

## Methods

### Participants

The participants were 24 preterm babies (14 girls and 10 boys). They were selected from intensive and regular neonatal care units in CHU of Grenoble. Parents gave written consent for their baby to participate in the experiment. The selection criteria were that they (i) had to show the grasp reflex, (ii) were not affected by a polymalformative syndrome, (iii) had to have a normal cranial ultrasonography, (iv) had to receive no sedative or anticonvulsive treatment during the experiment, and (v) had to be awake during tests. At birth, the mean gestational age was 30 weeks and 6 days (range from 26+3 to 34 weeks) and the mean weight was 1498 g. (range from 680 to 2723 g.). When the preterm babies were tested, the mean post-conceptional age ranged from 33 to 34+6 weeks, the mean post-natal age was 474 hours (range from 72 to 1200 hours), so about 20 days, and the mean weight was 1670 g. (range from 1000 to 2590 g.). It should be noted that the current World Health Organization definition of prematurity is a baby born before 37 weeks of gestation, counting from the first day of the last menstrual period, knowing that 40 weeks of gestation is the normal term. Moreover, viability of fetuses is between 22 and 24 weeks of gestation, depending on the country. The present study was conducted in accordance with the Declaration of Helsinki and approved by the local ethic committee of the LPNC (CNRS and University of Grenoble 2). The experiment was classified as purely behavioral, and the testing involved no discomfort or distress to the infants.

Equal numbers of the 24 participants were randomly assigned to the main Experimental (different stimulus object in the test phase) versus Control (same object in test as in the prior habituation phase) factors. In to ascertain the effects of two subsidiary stimulus (prism versus cylinder) and holding hand (right versus left) factors, the 24 participants were also divided into the relevant 4 groups of 6.

Furthermore, eight features of the medical history were collected in order to verify whether they could affect babies' performance in the habituation and test phases: 1) mode of delivery (vaginal delivery/caesarean section), 2) twin (yes/no), 3) antenatal steroids (yes/no), 4) hypotrophy (yes/no), 5) intubation (yes/no), 6) Continuous Positive Airway Pressure (yes/no), 7) nasal cannula for oxygenotherapy (yes/no) and 8) intravenous catheter (yes/no).

Table 1 presents the main results of the parameters of performance during both phases according to the medical history. Student's t-tests were performed to compare premature babies' performance for each medical feature (8) and for each parameter measured during the experiment (4). Because of the important number of comparisons (N = 32), a Bonferroni alpha-level correction was adopted (α = 0.05/32 = 0.001). Indeed, this correction avoids a lot of spurious positives with a decrease of the alpha value [22]. There was no significant difference between any of these values (all *p*>0.025). Thereby, medical history of preterm babies did not influence the performance measured during habituation and test phases. These results allowed us to carry out the next statistical analysis.

**Table 1:** Total holding times, holding times for the first two trials, mean number of trials of the habituation phase, and holding times for the two consecutive trials of the test phase (means (SD)) according to the eight features of the medical history.

				Habituation phase		Test phase
Category	Subcategory	N	Total holding time (sec.)	First two trials (sec.)	Number of trials	Mean holding time (sec.)
Mode of delivery	caesarean section	11	78.2 (34.8)	61.1 (35.8)	4.8 (1.1)	11.9 (10.3)
	vaginal delivery	13	81.3 (58.2)	53.4 (26.8)	4.3 (0.6)	17.6 (11.8)
Twin	yes	11	72.3 (43)	57.1 (34.3)	4.4 (0.7)	15.9 (9.6)
	no	13	86.3 (52.6)	58 (30.5)	4.7 (1)	14.2 (12.8)
Antenatal steroids	yes	21	84.6 (48.7)	60.1 (32)	4.6 (0.9)	15.2 (11.4)
	no	3	46.8 (27.9)	40.4 (26.5)	4 (0)	14 (12.6)
Hypotrophy	yes	4	71.3 (38.3)	48.1 (17.4)	4.5 (0.6)	8.4 (5.1)
	no	20	81.6 (50.3)	59.5 (33.7)	4.6 (0.9)	16.3 (11.8)
Intubation	yes	11	93.9 (60.1)	71.5 (38.6)	4.5 (0.8)	20.3 (12.6)
	no	13	68 (32.5)	45.9(18.4)	4.6 (1)	10.6 (8.1)
CPAP	yes	19	85.3 (50)	60.8 (33)	4.6 (1)	15.4 (12.1)
	no	5	59 (35.7)	45.5 (24.5)	4.2 (0.4)	13.7 (8.2)
Nasal cannula	yes	11	79.9 (43.6)	64.3 (36.3)	4.4 (0.7)	15.9 (11.3)
	no	13	79.8 (53)	51.9 (27.1)	4.7 (1)	14.2 (11.7)
Intravenous catheter	yes	16	86.3 (52.5)	60.6 (31.5)	4.6 (0.8)	17.3 (10.7)
	no	8	67.1 (37)	51.5 (32.9)	4.4 (1.1)	10.4 (11.2)

*(CPAP: Continuous Positive Airway Pressure).*

### Stimuli

**Figure 1:**
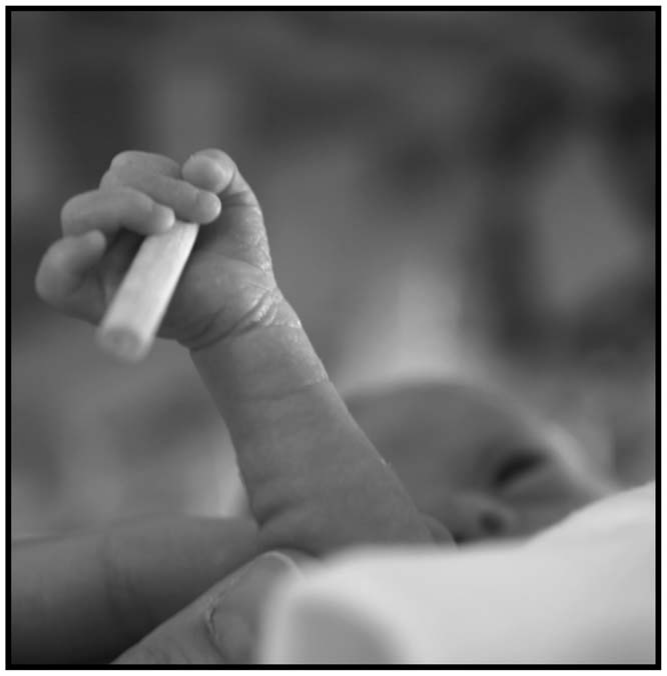
Preterm baby holding a cylinder (the baby's apparent visual fixation of the test hand can be discounted because the experimenters monitored head and eye movements).

**Figure 2:**
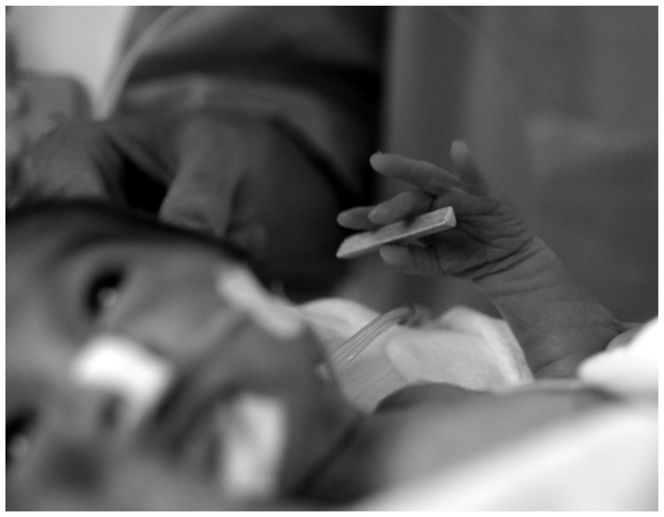
Preterm baby holding a prism.

The stimuli were a cylinder (a smoothly curved shape) and a prism (a sharply angled shape). These objects were chosen, because they elicit the grasp reflex, and are perfectly discriminated by full-term newborns' right and left hands [13]. The cylinder was 35 mm long and 6 mm in diameter and the prism was 35 mm long and 9x6x6 mm triangle base (see Figures 1 and 2). These objects were smaller than those used by Streri *et al*. (2000) [13], because preterms' hands were smaller than full-terms' hands. It is the surface ratio object/hand which is identical.

### Procedure

The preterm baby was tested in his incubator just before or just after his care, in an arousal state 4 of the Brazelton scale [23] and more than one hour after his alimentation. The tested hand had to be free (no scope and no perfusion). The first experimenter, a neonatologist, installed the baby in a semi-upright position during the whole experiment and positioned his head in the opposite side of the tested hand, so that the baby could not see the test object. The second experimenter, a psychologist, recorded holding times of objects with a hand-held computer which calculated a rate of habituation for each baby trial after trial. The whole experiment was videotaped to be analysed subsequently in order to verify and correct (when it was necessary) the holding times recorded by the hand-held computer.

#### Habituation phase

The first experimenter put an object in either the infant's right or left hand and the first trial started. The experimenter had to hold the preterm's forearm in order to cope with hypotonia (reduced muscular tonus). When the newborn grasped the object, the second experimenter began recording the holding time. When the premature babies released the object after holding it for at least 1 sec., the experimenter stopped the recording to end the trial. If the premature babies held the object for 60 sec., the first experimenter gently opened the infant's hand and removed the object, thus ending the trial. After an inter-trial interval of about 10 sec., the experimenter presented the object again, beginning another set of habituation trials. Our criterion for habituation was based on the two consecutive trials that followed the third trial. It required that the total holding time for the two following consecutive trials should last for not more than a third (or less) of the holding time for the first two trials, as in the previous study of full-term infants [13]. If our criterion of habituation was not met by the 12th trial, the infant was excluded from the experiment. Two groups were made according to the hand in which the object was put during the habituation phase, and then two subgroups were made according to the object's shape used during the same phase. The babies were randomly assigned to these four groups. Each group included six premature babies.

We used 4 measures in the habituation phase: holding times for the first two trials, total holding time until the criterion was reached, holding times for the last two habituation trials, and number of trials conducted.

#### Test phase

Then, the test phase could begin. In the test phase, the 12 Experimental (new object in test) and the 12 Control (same object in test) group subjects received two trials. The experimenter placed the relevant (same or different) test object in the same hand of the baby as in the habituation phase.

An important measure in the test phase was the mean holding time for the two consecutive trials. We defined discrimination as having occurred when the mean holding time for the novel object was greater than the mean holding time displayed in the last two habituation trials. On the contrary, we expected that the mean holding time for the familiar object and the mean holding time displayed in the last two habituation trials not to differ significantly. The measure was used here to test whether preterm newborns do - or do not - discriminate sharp from smooth shape features by touch.

### Design

For the habituation phase, statistical analyses were performed with two main factors: a between-subjects factor (Group: experimental vs. control) × a within-subjects factor (Trials: first two habituation trials vs. last two habituation trials). For the test phase, statistical analyses were performed with two main factors: a between-subjects factor (Group: experimental vs. control) × a within-subjects factor (Phase: last two habituation trials vs. two test trials). Moreover, to ascertain whether the main data were affected by laterality or type of shape features, subsidiary statistical analyses were performed with two between-subjects factors: Hand (Left vs. Right) and Shape (Cylinder vs. Prism). Finally, to compare performance between preterm and full-term newborns, an additional statistical analysis was performed with a between-subjects factor: Population Type (preterm vs. full-term).

## Results

Results are reported in 2 subsections: (1) Findings for preterm newborns in (a) the habituation and (b) the test phase, and (2) results of comparing these findings with previous findings for full-term newborns in both phases.

### 1. Findings for Preterm Newborns

#### a. Habituation phase

A 2 (Group: experimental vs. control) ×2 (Trials: first two habituation trials vs. last two habituation trials) ANOVA was performed for the holding times. The results showed a significant effect of the Trials factor (F(1, 22) = 66.523; p<0.001): preterm babies held significantly longer the object during the first two habituation trials (Mean (M) = 57 s) than during the last two habituation trials (M = 8 s). There were no significant effect for the Group factor (F(1, 22) = .544; p = .469) and for the Trials × Group Interaction (F(1, 22) = .075; p = .786). It indicated that a successful and similar tactile habituation occurred for both experimental and control groups.

See Table 2 for total holding times, holding times for the first two trials, and number of trials of habituation for both objects and both hands. To ascertain whether the habituation measures were affected by laterality or type of shape features, a 2 (Hand: right vs. left) ×2 (Shape: prism vs. cylinder) ANOVA was performed. First, for the mean total holding times, there were no significant effect of the Hand factor (*F*(1, 23) = .492; *p* = .491), Shape factor (*F*(1, 23) = .347; *p* = .563) and Hand × Shape Interaction (*F*(1, 23) = .008; *p* = .928). Second, for the mean holding times of the first two trials, there were no significant effect of the Hand factor (*F*(1, 23) = .003; *p* = .956), Shape factor (*F*(1, 23) = .290; *p* = .596) and Hand × Shape Interaction (*F*(1, 23) = 1.454; *p* = .242). Finally, for the mean number of trials, there were no significant effect of the Hand factor (*F*(1, 23) = 2.952; *p* = .101), Shape factor (*F*(1, 23) = .060; *p* = .809) and Hand × Shape Interaction (*F*(1, 23) = 2.952; *p* = .101). In conclusion, the effects of these factors and interactions were not significant (*all p*>0.10), indicating in particular that both objects are equally graspable by preterm infants' right and left hands.

**Table 2:** Total holding times, holding times for the first two trials, and number of trials during habituation (means and (SD)) according to the hand (right or left) and to the object (prism or cylinder).

Hand	Object	Total holding time (sec.)	First two trials (sec.)	Number of trials
Right hand	Prism (N = 6)	65.7 (30.1)	45.7 (18.6)	4.5 (1.2)
	Cylinder (N = 6)	79.7 (45.3)	68.8 (39.2)	4 (0)
Left hand	Prism (N = 6)	82 (44.8)	62.4 (31.8)	4.5 (0.6)
	Cylinder (N = 6)	92.2 (71.9)	53.5 (36.2)	5.2 (1)

Finally, table 3 presents Bravais-Pearson correlations between 3 habituation measures (mean total holding times, mean holding times for the first two trials, and number of trials) and gestational age, post-natal age, post-conceptional age, birth weight and weight at test. There was no significant correlation between all these factors (*all p*>0.10).

**Table 3:** Bravais-Pearson correlations (Pearson's *r* and p value) between total holding times, holding times for the first two trials, mean number of trials of the habituation phase, holding times for the two consecutive trials of the test phase and gestational age, post-natal age, post-conceptional age, birth weight and weight at test.

Phase	Measures	Gestational age	Post-natal age	Post-conceptional age	Birth weight	Weight at test
Habituation	Total holding time	−.007	0	−.106	−.244	−.354
		**p = .976**	**p = 1**	**p = .621**	**p = .250**	**p = .101**
	First two trials	−.089	.099	−.083	−.219	−.220
		**p = .678**	**p = .646**	**p = .699**	**p = .303**	**p = .303**
	Number of trials	.076	−.091	−.041	−.163	−.327
		**p = .725**	**p = .671**	**p = .849**	**p = .447**	**p = .119**
Test	Mean holding time of	−.078	.094	.084	.010	.037
	the two test trials	**p = .715**	**p = .661**	**p = .698**	**p = .962**	**p = .863**

#### b. Test phase

A 2 (Group: experimental vs. control) ×2 (Phase: last two habituation trials vs. two test trials) ANOVA was performed for the holding times. Results showed no significant effect of the Group factor (F(1, 22) = 1.410; p = .248), and a significant effect of the Phase factor (F(1, 22) = 27.044; p<0.001) explained by a significant Phase × Group Interaction (F(1, 22) = 5.458; p = 0.029).

To investigate this Phase × Group interaction further, planned comparisons were performed: the experimental group held significantly longer the novel object (*M* = 18.9 s) compared to the last two habituation trials (*M* = 3.4 s) (*F*(1, 22) = 28.4; *p*<0.001). This suggests that a reaction to a novel shape is obtained in preterm babies. For the control group, babies held the familiar object during the test phase (*M* = 11.1 s) as much as during the last two habituation trials *(M* = 5.3 s) (*F*(1, 22) = 4.10; *p* = 0.055).

The above difference in holding time for the control group approached significance although it was the same object in the two phases. The analysis of individual data allows us to understand this surprising result. Table 4 presents individual data for each preterm baby and shows a baby (S24) “blocking” on the familiar object in the test phase (holding time  = 60 s). We had to remove it although he was considered as habituated to this object. We supposed that this behavior could be responsible for this unexpected difference. In order to test this assumption and to homogenize our approach across groups, we substituted three data equal to 60 seconds (one in control group -S24- and two in experimental group -S1 and S11-) by the mean of holding times of subjects who were in the same experimental conditions. Table 4 presents the modified data in brackets. Then, a similar 2 (Group: experimental vs. control) ×2 (Phase: last two habituation trials vs. two test trials) ANOVA was performed for these modified data. The analyses confirmed a significant Phase × Group Interaction (*F*(1, 22) = 4.242; *p* = 0.05) and planned comparisons now showed that the experimental group held the novel object longer (*M* = 15.11 s) compared to the last two habituation trials (*M* = 3.4 s) (*F*(1, 22) = 18.55; *p*<0.001), whereas the difference of mean holding times was not significant for the control group anymore (*F*(1, 22) = 1.94; *p*>0.15). These results are consistent with the explanation that the unexpected difference was due to the blocking behavior of one baby.

**Table 4:** Holding times displayed in the last two habituation trials and their mean holding time, and holding times of test trials 1 and 2 and means of the two test trials for each participant (S) and each group (experimental vs. control).

			Habituation Phase			Test Phase	
Group	S	Holding Time (sec.) Last habituation trial 1	Holding Time (sec.) Last habituation trial 2	Mean holding time (sec.)	Holding Time (sec.) Test trial 1	Holding Time (sec.) Test trial 2	Mean holding time (sec.)
Experimental	1	1.262	1.199	1.23	1.257	*60 (27.3675)*	*30.63 (14.31)*
(N = 12)	2	3.192	1.787	2.49	8.164	20.783	14.47
	3	1.559	4.019	2.79	14.81	33.952	24.38
	4	5.94	2.402	4.17	50.557	1.082	25.82
	5	2.234	11.307	6.77	3.807	2.534	3.17
	6	1.755	9.612	5.68	34.75	18.792	26.77
	7	1.208	1.22	1.21	11.093	18.024	14.56
	8	8.248	1.652	4.95	19.445	6.632	13.04
	9	2.98	1.05	2.02	21.756	1.708	11.73
	10	1.293	2.428	1.86	53.655	2.404	28.03
	11	2.851	4.442	3.65	2.321	*60 (2.1685)*	*31.16 (2.24)*
	12	6.793	1.681	4.24	3.652	1.933	2.79
M (modified M)		**3.28**	**3.57**	**3.42**	**18.77**	**18.99 (11.44)**	**18.88 (15.11)**
Control	13	2.75	4.783	3.77	1.148	1.144	1.15
(N = 12)	14	4.414	3.215	3.81	2.496	17.796	10.15
	15	9.264	3.116	6.19	18.117	1.981	10.05
	16	2.037	6.021	4.03	5.155	2.276	3.72
	17	1.95	9.633	5.79	2.801	1.465	2.13
	18	3.303	3.09	3.2	1.819	9.964	5.89
	19	4.017	1.878	2.95	3.475	17.334	10.4
	20	1.684	7.06	4.37	9.399	1.754	5.58
	21	7.157	5.24	6.2	8.925	58.998	33.96
	22	2.193	1.686	1.94	1.388	4.773	3.08
	23	3.993	6.397	5.2	18.282	8.35	13.32
	24	2.088	29.37	15.73	*60 (9.835)*	8.473	*34.24 (9.15)*
M (modified M)		**3.74**	**6.79**	**5.27**	**11.08 (6.9)**	**11.19**	**11.14 (9.05)**

*In italics: data to substitute; in italics and brackets: modified data; in bold: mean holding times for each group.*

To ascertain whether the holding times during test phase were affected by laterality or type of shape features, a 2 (Hand: right vs. left) × 2 (Shape: prism vs. cylinder) ANOVA was performed. Results showed that Hand factor (*F*(1, 23) = .108; *p* = .747), Shape factor (*F*(1, 23) = .600; *p* = .450) and Hand × Shape Interaction (*F*(1, 23) = .607; *p* = .447) did not influence the holding times during test phase.

Finally, table 3 presents Bravais-Pearson correlations between holding times for the two consecutive trials of the test phase and gestational age, post-natal age, post-conceptional age, birth weight and weight at test. There was no significant correlation between all these factors (*all p*>0.25), suggesting that gestational age, post-natal age, post-conceptional age, birth weight and weight at test did not influence holding times during test phase.

### 2. Results of Comparing These Findings with Previous Findings for Full-Term Newborns in Both Phases

Data from Streri *et al*.'s study (2000) were compared with our data in order to investigate if performance were different between preterm and full-term newborns.

#### Habituation phase

See Table 5 for mean total holding times, mean holding times for the first two trials, and number of trials, respectively, for preterm and full-term newborns. We used Student's t-tests to compare the respective means of all three measures for the two populations. The total holding time for preterm babies was significantly shorter than for full-term babies (t(46) = −2.256; p = 0.029). The mean holding time for the first 2 trials was longer for preterms than for full-terms, but not significantly so (t(46) = 1.609, p = .173). Preterms took significantly fewer trials to reach the habituation criterion than full-term babies (t(46) = −4.932; p<0.001).

**Table 5:** Comparison of total holding times, holding times for the first two trials, and number of trials of habituation (means and (SD)) between preterm and full-term newborns.

Population Type	Total holding time (sec.)	First two trials (sec.)	Number of trials
Preterm (N = 24)	79.9 (47.9)	57.6 (31.5)	4.5 (0.9)
Full-term (N = 24)	123.1 (63.7)	45.9 (27)	6.4 (1.6)
t	−2.256 *	1.609	−4.932 ***

t indicates the result of t-tests (*p<.05 and ***p<.001).

#### Test phase

Table 6 shows the mean holding times during test phase between preterm and full-term newborns. Regarding the full-term newborns' data in the test phase, we used holding times of the two test trials of the novel object for the “non-lag group” (label from Streri et al.'s study (2000)), because this “non-lag group” was exactly in the same conditions as our experimental group. Otherwise, the “lag group” received, after habituation, two test trials with the familiar object following by two additional test trials with the novel object, but we used only mean holding times of the two test trials of the familiar object for the current comparison, in order to be in the same conditions as our control group. A 2 (Population Type: preterm vs. full-term) ×2 (Group: experimental vs. control) ANOVA was performed for holding times for the two consecutive trials of the test phase. The analyses revealed a main effect of Group (F(1, 47) = 9.806; p = 0.003), confirming a novelty reaction for the new shape for both full-term and preterm babies. Indeed, the experimental group held the novel object (M = 19 s) longer than the control group with the familiar object (M = 9 s). However, neither main effect of Population Type (F(1, 47) = 0.384; p>0.25) nor interaction Population Type × Group (F(1, 47) = 0.496; p>0.25) were observed.

**Table 6:** Mean holding times during test phase (means and (SD)) in preterm and full-term newborns.

Population Type	Group	Holding time (sec.)
Preterm (N = 24)	Control	11.14 (11.3)
	Experimental	18.88 (10.2)
Full-term (N = 24)	Control	6.9 (5.4)
	Experimental	19.1 (15)

## Discussion

This study investigated the ability of preterm babies' hands to perceive the difference between two shapes and revealed three main results. Firstly, when an object was put in the preterm newborns' hand, the holding time decreased trial after trial until the habituation criterion was reached. For the first time, the results reveal that a haptic manual habituation is present for each preterm newborn between 33 and 34+6 GW. This result is consistent with Fearon, Hains, Muir and Kisilevsky's study (2002) about passive touch [24]. The authors showed that the majority of preterm infants between 30 and 36 GW displayed tactile habituation: in active sleep and the stimulus was a gentle stroke on the infant's forearm twice from wrist to elbow done by the experimenter. In fact, successful habituation can be considered as an elementary kind of learning. Because habituation shares some links with memory and is thought to involve processes that reflect the development of some internal representation of a stimulus [25], it means that preterm babies are able to memorize the shape of an object with each hand.

Secondly, after habituation, when an object with a novel shape was put in the preterm newborns' hand, the holding time increased. This is the first evidence that preterm infants between 33 and 34+6 GW are capable of manual discrimination (active touch) between a prism and a cylinder, whichever hand tested. Consequently, the grasping at 33 GW would be not only a reflex because preterm babies could retain tactile information about specific shape features and detect the differences of shape when a novel stimulus is presented in the manual mode.

Finally, data from Streri *et al*'s study (2000) [13] were compared with the present data in order to investigate whether these perceptual manual abilities were qualitatively and/or quantitatively different between preterm and full-term newborns. They provided evidence for manual haptic habituation and discrimination with either right or left hands in full-term newborns and we observed similar results in preterm babies. This suggests that these perceptual manual abilities were qualitatively similar but are they quantitatively similar too? The comparison revealed that preterm babies habituated more quickly whereas the performance during the test phase did not differ significantly. The length of the habituation time observed in this study could be affected by motor fatigue that is a well known phenomenon in preterm babies [26]. But, this result can suggest that the observed habituation reveals a mere motor fatigue. If this was the case then discrimination between shapes had not to be observed. This faster habituation in preterm babies seems to be sufficient and efficient to lead to a reaction to the novel object. So, this motor fatigability would not disturb the habituation ability in preterm babies. Taken together, these results suggest that the processes involved in the manual habituation and discrimination of shapes are only qualitatively similar between preterm and full-term newborns.

In short, a manual habituation occurs in preterm babies between 33 and 34+6 GW. Then, in this case, a manual discrimination of shapes is found in preterm babies, whatever the hand. These results are consistent with those observed in full-term newborns. However, a difference in the speed habituation process exists between preterm and full-term babies. It probably indicates mainly a difference of motor maturation: preterm infants tire more quickly when holding an object. The results suggest that there is no qualitative–but only a quantitative- difference between the perceptual abilities of preterm babies' hand and those of full-term babies. Further studies are needed to address the question of higher cognitive functions like intermanual transfer [27] and intermodal transfer between touch and vision in preterm newborns [28], [29], [30], [31].
